# Editorial: Maternal Health in Conflict Settings

**DOI:** 10.3389/fgwh.2022.807257

**Published:** 2022-03-10

**Authors:** Ribka Amsalu, Tabassum Firoz, Isabelle L. Lange, Hannah Tappis

**Affiliations:** ^1^California Preterm Birth Initiative, University of California, San Francisco, San Francisco, CA, United States; ^2^Department of Obstetrics, Gynecology and Reproductive Sciences, Medical Center, University of California, San Francisco, San Francisco, CA, United States; ^3^Department of Medicine, Yale School of Medicine, New Haven, CT, United States; ^4^Department of Infectious Disease Epidemiology, London School of Hygiene and Tropical Medicine, London, United Kingdom; ^5^Department of Global Health and Development, London School of Hygiene and Tropical Medicine, University of London, London, United Kingdom; ^6^Jhpiego, Baltimore, MD, United States

**Keywords:** maternal health, conflict, maternal mortality, maternal morbidities, refugee, humanitarian emergencies

The Sustainable Development Goals (SDGs), endorsed in 2015 by the United Nations, aim to reduce the global maternal mortality ratio to less than 70 per 100,000 live births by 2030 (1). The world is not on track to achieve this goal. The Ending Preventable Maternal Mortality (EPMM) initiative, which includes a broad coalition of partners working in maternal health, has established five targets and ten milestones that need to be achieved by 2025 if the SDGs are to be met (2). Though the EPMM milestones include preparedness, response and resilience to emergencies, the challenge is more acute in humanitarian settings.

The upsurge in violent conflicts and displacement around the world in the last two decades has given rise to more than 70 million displaced persons, and instability and conflict in several countries (3). By 2017, conflict-affected and fragile countries had the worst maternal mortality ratio per 100,000 live births with estimates of 1,150 (95% CI: 789, 1,710) in South Sudan, 1,140 (95% CI: 847, 1,590) in Chad, 829 (95% CI: 463, 1,470) in the Central African Republic, 829 (95% CI: 385, 1,590) in Somalia, and 638 (95% CI: 427, 1,010) in Afghanistan (4). Implementation of health services and research on maternal health in conflict-affected countries is constrained by operational challenges (insecurity, remote locations, shortage and high turnover of staff), lack of investment, and competing priorities (5, 6). These challenges are exacerbated by the added burden of the COVID-19 pandemic, Ebola virus disease outbreaks, and lack of investment in research.

In the absence of up-to-date data, our capacity to monitor progress achieved in mitigating the impact of conflict on maternal survival and our ability to advocate for increases in investment to scale-up promising approaches is limited. The Frontiers Maternal Health in Conflict Settings special issue aimed to create a platform to highlight new research findings. Our call for papers was announced in March 2020 and received 14 article submissions. The final seven articles accepted in four key areas present research from eight countries and authors from academic institutions, non-governmental organizations, and intergovernmental agencies.

1. *Reliable data on maternal health service availability, utilization and outcomes is needed to inform public health action*. Maruf et al. provides sobering insights into the quality of health facility records in Afghanistan and the challenges that gaps in data availability and quality present for prioritization of health improvement. In contrast, the study by Chukwama et al. found how the impact of conflict on health service delivery can be illuminated when data are available. In one of the few studies that quantifies the impact in the African context, the authors found that even if infrastructure is not demolished following conflict, the quality of maternal health care may be negatively affected as women receive fewer than the recommended components of care.
2. *Comprehensive maternal health care through pregnancy, delivery, and postpartum is critical*. Gallagher et al.'s analysis of family planning data from the Democratic Republic of Congo, Pakistan, Rwanda, Somalia, Syria and Yemen illustrates the benefits of investing in immediate postpartum family planning interventions in conflict-affected areas and that uptake of contraception is high when a quality service is available. Tran et al.'s evaluation of family planning and postabortion care programs in conflict settings highlights the critical need to have health system components, engagement of national stakeholders, and a better understanding by donors and the international community that health system strengthening can be achieved and must be emphasized in the acute and protracted phases of a humanitarian response.
3. *Health worker and women's experiences, preferences, and utilization patterns matter*. Bashour et al. undertook a qualitative study among obstetricians and pregnant women at a teaching hospital in Damascus during the war in Syria between 2012 and 2014. They found effects of the conflict on both groups' access to health facilities, and, once there, providers' ability to deliver comprehensive, emotionally supportive, quality services. Though there was a sense of accomplishment that the hospital was able to continue to offer much needed care, the physical and mental trauma due to violence and uncertainty could not be discounted, and should be taken into consideration when planning maternal services during war. Health services in humanitarian contexts are generally studied in isolation; however, Rustad et al. provide a unique insight by comparing maternal health care between refugee and host communities in the West Nile region of Uganda as a way of identifying potential inequities. The authors found that, though care is sought from the same facilities, refugee populations were more likely to be less satisfied and believed they had been discriminated against during antenatal care, providing important findings for policy makers and administrators.
4. *Understanding the socio-economic determinants of who accesses and utilizes maternal health care services in conflict settings is needed to inform implementation strategy*. Kim et al. examined key socio-economic and demographic factors associated with women's place of birth preference in Afghanistan. They found that a combination of factors such as access to secondary education, adequate antenatal care, and motor vehicles, had the largest change on the predicted decrease in home births and increased the likelihood of in-facility births. The study highlights the need for multi-sectoral efforts, which is especially critical in the new political climate in Afghanistan.

Gaps in research remain in postpartum care, morbidities during pregnancy (hypertension, infection, diabetes), understanding the preferences and experiences of women, and in certain humanitarian crises. We also need to shift our thinking and our approach in the humanitarian health sector from concentrated power in Western institutes to academics, citizens' and advocacy groups, policy makers and implementers based in countries affected by conflict. As the world mobilizes resources to overcome the challenges of COVID-19 pandemic, we must ensure that women and their communities affected by conflict are included in global initiatives; such as the COVAX program for COVID-19 vaccines, mitigation efforts are taken to reduce maternal infection and the indirect impact of the pandemic on maternal health, and investment committed for research in conflict settings so that we can have better data to act on.

## Author Contributions

All authors listed have made a substantial, direct, and intellectual contribution to the work and approved it for publication.

## Funding

RA was supported by a University of California, San Francisco, Preterm Birth Initiative transdisciplinary post-doctoral fellowship, funded by Marc and Lynne Benioff and a T32 training grant (1T32HD098057) from the National Institute of Child Health and Human Development entitled Transdisciplinary Research Training to Reduce Disparities in Preterm Birth and Improve Maternal and Neonatal Outcomes.

## Conflict of Interest

The authors declare that the research was conducted in the absence of any commercial or financial relationships that could be construed as a potential conflict of interest.

## Publisher's Note

All claims expressed in this article are solely those of the authors and do not necessarily represent those of their affiliated organizations, or those of the publisher, the editors and the reviewers. Any product that may be evaluated in this article, or claim that may be made by its manufacturer, is not guaranteed or endorsed by the publisher.
